# An Optimized Direct Lysis Gene Expression Microplate Assay and Applications for Disease, Differentiation, and Pharmacological Cell-Based Studies

**DOI:** 10.3390/bios12060364

**Published:** 2022-05-26

**Authors:** Neville S. Ng, Simon Maksour, Jeremy S. Lum, Michelle Newbery, Victoria Shephard, Lezanne Ooi

**Affiliations:** 1 Illawarra Health and Medical Research Institute, Northfields Avenue, Wollongong, NSW 2522, Australia; sm107@uowmail.edu.au (S.M.); jlum@uow.edu.au (J.S.L.); mn631@uowmail.edu.au (M.N.); vshephar@uow.edu.au (V.S.); 2 School of Chemistry and Molecular Bioscience and Molecular Horizons, University of Wollongong, Northfields Avenue, Wollongong, NSW 2522, Australia; 3 School of Medical, Indigenous and Health Sciences, University of Wollongong, Northfields Avenue, Wollongong, NSW 2522, Australia

**Keywords:** microplate, gene expression, direct lysis, cell-based assay

## Abstract

Routine cell culture reverse transcriptase quantitative polymerase chain reaction (RT-qPCR) gene expression analysis is limited in scalability due to minimum sample requirement and multistep isolation procedures. In this study, we aimed to optimize and apply a cost-effective and rapid protocol for directly sampling gene expression data from microplate cell cultures. The optimized protocol involves direct lysis of microplate well population followed by a reduced thermocycler reaction time one-step RT-qPCR assay. In applications for inflammation and stress-induced cell-based models, the direct lysis RT-qPCR microplate assay was utilized to detect *IFN1* and *PPP1R15A* expression by poly(I:C) treated primary fibroblast cultures, *IL6* expression by poly(I:C) iPSC-derived astrocytes, and differential *PPP1R15A* expression by ER-stressed vanishing white-matter disease patient induced pluripotent stem cell (iPSC)-derived astrocytes. In application for neural differentiation medium recipe optimizations, conditions were screened for *SYN1* and *VGLUT1* in neuronal cultures, and *S100B*, *GFAP* and *EAAT1* in astrocyte cultures. The protocol provides microplate gene expression results from cell lysate to readout within ~35 min, with comparable cost to routine RT-qPCR, and it may be utilized to support laboratory cell-based assays in basic and applied scientific and medical fields of research including stem-cell differentiation, cell physiology, and drug mechanism studies.

## 1. Introduction

Gene expression by reverse transcription quantitative polymerase chain reaction (RT-qPCR) is routinely implemented in medical research and clinical diagnostic laboratories. RT-qPCR generally involves four main stages: nucleic acid isolation, genomic DNA removal, complementary DNA synthesis (cDNA), and quantitative PCR. Routine laboratory RNA isolation involves biphasic layer separation to separate RNA and protein, followed by ethanol precipitation [1] or column binding of RNA to silica membrane in high salt concentrations, with washing and low-salt volume elution [2]. Recently, RT-qPCR methods with direct specimen lysis to circumvent RNA isolation methods have been demonstrated [3]. Although one-step RT-qPCR products that combine cDNA synthesis and qPCR stages are commercially available, the cost of commercial direct lysis kits is significantly higher than that of conventional RT-qPCR methods and prohibitive to large-scale experiments. As such, we aimed to develop and implement a comparatively cost-effective and rapid protocol from cell lysis to results in microplate format. Direct lysis gene expression studies have previously implemented buffers based on hypotonic deionized water, Igepal CA-630 and BSA [3,4], and Tris-NaCl Igepal CA-630 or Triton X-100 [5], for the detection of low-population circulating tumor cell studies [3] and single-cell gene expression profiling of primary mouse astrocytes [4]. In this study, a direct lysis extraction protocol utilizing a one-step TaqMan RT-qPCR assay with reduced cycle time and reaction volumes, at reduced cost relative to commercial direct lysis RT-qPCR products, was optimized for microplate application. The protocol can obtain gene expression data from endpoint to analysis within 35 min and allow samples to be stored at −80 °C for at least several months [5]. The method was implemented in human dermal fibroblasts, as well as induced pluripotent stem-cell-derived astrocytes and neurons, for the detection of differentiation, cell stress, and inflammatory gene expression modulation. The protocol can be utilized for gene expression characterization in tandem with other cell-based assays, and can be economically scaled toward higher-throughput applications. Consequently, this method can be used to support disease modeling or drug discovery.

## 2. Materials and Methods

### 2.1. Cell Culture

All experimental protocols were approved by the University of Wollongong Human Research Ethics Committee (HE17/522). The iPSCs were previously generated and characterized [6]. Fibroblasts were maintained in 5% CO_2_ at 37 °C. All other cell types were maintained in hypoxic conditions with 5% CO_2_ and 3% O_2_ at 37 °C. Human primary fibroblasts were maintained in DMEM/F12 (21331020, Thermo Fisher Scientific, Waltham, MA, USA), 10% fetal bovine serum (FBS) (SFBS-AU, Bovogen Biologicals, Keilor, Australia), 2 mM GlutaMAX (35050079, Thermo Fisher Scientific, Waltham, MA, USA), and 0.5% penicillin/streptomycin (15140122, Thermo Fisher Scientific, Waltham, MA, USA).

### 2.2. Differentiations

Human neural precursor cells (NPCs) were generated by dual SMAD inhibition with incubation of induced pluripotent stem-cell cultures with ALK inhibitors, 0.1 µM LDN193189 (HY-12071A-10MG, Focus Bioscience, Murarrie, Australia) and 10 µM SB431542 (HY-10431-10MG, Focus Bioscience, Murarrie, Australia) in neural medium (2% B27 supplement (17504001, Thermo Fisher Scientific, Waltham, MA, USA) and GlutaMAX in DMEM/F12) for 5 days, followed by replacement of small molecules with 20 ng/mL epidermal growth factor (EGF) (130-097-749, Miltentyi Biotec GmbH, Bergisch Gladbach, Germany) and 20 ng/mL basic fibroblast growth factor (FGF-2) (130-093-841, Miltentyi Biotec GmbH, Bergisch Gladbach, Germany) (Figure S1) [6].

Neuronal differentiation was driven by a lentiviral vector encoding the transcription factor, neurogenin-2 (NGN2), as similarly described in [7,8,9,10]. Briefly, NPCs were transduced with viral particles overnight before gene expression was induced by exposure to 2 µg/mL doxycycline (D3447-500MG, Sigma-Aldrich, St. Louis, MO, USA) for 3 days and selection with 2 µg/mL puromycin for 3 days after the first day of doxycycline induction (P8833-10MG, Sigma-Aldrich, St. Louis, MO, USA) in neuronal medium (DMEM/F12, 2 mM GlutaMAX, 2% B27, 10 ng/mL brain derived neurotrophic factor (BDNF) (130-096-286, Miltentyi Biotec GmbH, Bergisch Gladbach, Germany), 5 µM SU5402 (HY-10407-10MG, Focus Bioscience, Murarrie, Australia), 5 µM DAPT (HY-13027-10MG, Focus Bioscience, Murarrie, Australia), and 500 µM dibutyl cyclic adenosine monophosphate (db-cAMP) (D0627-1G, Sigma-Aldrich, St. Louis, MO, USA). Astrocytes were differentiated by transduction of NPCs driven by lentiviral particles driving overexpression of SRY-Box Transcription Factor 9 (SOX9) and Nuclear Factor I B (NFIB), as similarly described [11]. 

Neuronal and astrocyte cell cultures were analyzed during days 10–14 of differentiation (Figure S1). The eIF2B/vanishing white-matter (VWM) disease astrocytes were differentiated and maintained by a previously described growth factor-directed differentiation method and analyzed during weeks 6–8 [6], generated from NPCs incubated in a ciliary neurotrophic factor (CNTF)-based medium for 4 weeks before incubation in DMEM/F12, 2% FBS, and Astrocyte Growth Supplement (1852, Sciencell, Carlsbad, CA, USA).

### 2.3. Inflammatory, ER Stress, and Neural Differentiation Assays

Polyinosinic–polycytidylic acid (poly(I:C)) (P1530, Sigma-Aldrich, St. Louis, MO, USA) was prepared at 1 mg/mL in 0.9% *w*/*v* saline and reannealed by heating at 70 °C for 10 min before cooling at ambient temperature for 1 h, as previously described [12]. Inflammation stress response assays were conducted by incubation of astrocytes with poly(I:C) at 10 µg/mL or fibroblasts overnight with 0.1–1000 ng/µL poly(I:C). ER stress assays with VWM disease lines were performed as previously described with overnight incubation of 0.1 µM MG132 [6]. For neuronal maturation small-molecule assays, NPCs transduced with NGN2 were incubated with combinations of 5 µM DAPT, SU-5402, Y-27632, 10 ng/mL BDNF, or BDNF and glial-derived neurotrophic factor (GDNF). Functional activity of glutamate activity in neurons was assessed by the ratio of F_340/380_ of neuronal of cultures preincubated with 3 µM Fura-2 AM (F1221 Thermo Fisher Scientific, Waltham, MA, USA) for 0.5 h, in response to 50 µM glutamic acid, as analyzed by fluorescence spectroscopy (Molecular Devices Flexstation 3) for 7–10 days. For astrocyte differentiation factor assays, NPCs transduced with SOX9–NFIB were incubated with 100 ng/mL CNTF (130-108-972 Miltenyi Biotec GmbH, Bergisch Gladbach, Germany), BMP4 (130-111-165 Miltenyi Biotec GmbH, Bergisch Gladbach, Germany), FGF-2, EGF, 0.5 mM DBC, or 2% FBS for 7 days.

### 2.4. Direct Lysis RT-qPCR

For column extraction controls, RNA was extracted from cell cultures using the PureLink RNA mini column isolation (12183025, Thermo Fisher Scientific, Waltham, MA, USA) and on-column genomic DNA removal with PureLink DNase (12185010, Thermo Fisher Scientific, Waltham, MA, USA). Direct lysis was performed with a 200 µL 0.9% *w*/*v* saline wash followed by lysis in 5 mM Tris, 75 mM saline, and 0.05% Triton X-100 for 5 min, before storage at −80 °C.

### 2.5. RT-qPCR

Gene expression experiments were conducted with an Applied Biosystems Scientific QuantStudio 5. RT-qPCR reactions were carried out in 10 µL volumes in MicroAmp 96-Well Reaction Plates (4366932, Thermo Fisher Scientific, Waltham, MA, USA). Each target or multiplex combination was prepared with TaqPath One-Step RT-qPCR Master Mix (A15300, Thermo Fisher Scientific, Waltham, MA, USA). Standard thermal cycling conditions were performed with reverse transcriptase at 50 °C for 15 min (1×), enzyme activation at 95 °C for 2 min (1×), and amplification 95 °C for 3 s, followed by 60 °C for 15 s (40×) (Applied Biosystems QuantStudio 5). TaqMan predesigned assays (exon junction spanning) included Hs00174131_m1 IL6, Hs00169585_m1 PPP1R15A, Hs00389217_m1 S100B, Hs00909233_m1 GFAP, Hs00904823_g1 SLC1A3, Hs00220404_m1 SLC17A7, Hs00199577_m1 SYN1, Hs02758991_g1 GAPDH, Hs02800695_m1 HPRT1 (4331182, Thermo Fisher Scientific, Waltham, MA, USA), and Hs00427620_m1 TBP (4448489, Thermo Fisher Scientific, Waltham, MA, USA).

### 2.6. Statistical Analysis

All reactions were performed with technical duplicate wells, three independent RT-qPCR experiments for thermocycler experiments, and three biological replicates for all other experiments. Statistical analyses were performed using GraphPad Prism 8. Comparisons between groups were performed with Student’s *t*-test (2 groups) or one-way ANOVA followed by Holm–Sidak post hoc analysis (multiple groups).

## 3. Results and Discussion

**Figure 1:**
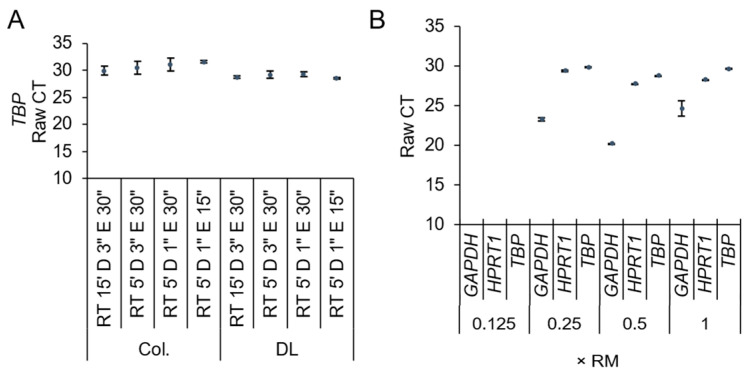
Optimization of thermocycler time and reaction mix concentration. (**A**) Decreasing reverse transcriptase (RT), denaturation (D), and elongation (E) phase times did not significantly affect the CT of the housekeeper gene (*TBP*) with RNA extracted by column (Col.) or direct lysis (DL) extraction methods. (**B**) Minimizing reaction reagent mix concentration (RM) as low as 0.25–0.5× did not significantly affect the CT of the housekeeper gene (one-way ANOVA followed by Holm–Sidak post hoc multiple comparison, *n* = 3).

The thermocycler reaction time and reaction mixture of the TaqMan 1-step RT-qPCR assay kit were optimized to establish a rapid and routine method to analyze the gene expression of cell cultures in microplate format. Although increasing qPCR elongation time is known to increase yield of product, the maximal elongation rate of Taq polymerase (150 bp/s at 75 °C) indicates that typical thermal cycling stages could be greatly decreased [13]. Prior studies have indicated an elongation thermal cycler duration as low as 5 s without spurious results; however, due to equipment limitations in heating ramp rate, this is not necessarily feasible on common RT-qPCR machines. Reduced denaturing and elongation times during qPCR amplification do not negatively impact amplification yield [13,14]; thus, an elongation phase of 15 s was used. The reverse transcriptase duration was also decreased on the basis of the typical operation rate of Moloney Murine Leukemia Virus (MMLV) (>10 NT/s) [15]. No significant differences in raw threshold cycle (CT) values were observed with decreases in reverse transcriptase, denaturing time, or elongation time (Figure 1A). Overall, this reduced the overall cycle time from 50 min to 30 min.

To increase reliability and compatibility with higher-throughput liquid handling, as well as reduce low-volume pipetting error [16], a simple two-step liquid transfer of template from the cell lysis buffer and reaction master mix, using 5 µL rather than 1 µL template volumes, was utilized. However, with a previously reported Tris-buffered saline direct lysis buffer [3], this increase in template volume was initially found to be inhibitory to reverse transcriptase or DNA amplification in comparison to template diluted in water alone (Figure S2). The direct lysis buffer that was not inhibitory to RT or qPCR amplification of any gene targets was composed of reduced NaCl (75 mM), with 5 mM Tris and 0.05% Triton X-100. At this saline concentration the buffer osmotic concentration was hypotonic (~315 reduced to ~130 mOsm/kg), which may have assisted with cell membrane rupture, similar to deionized water lysis reactions. Within 5 min of exposure, a flattened morphology and a high decrease in cellular protein staining but intact nuclear staining was observed (Figure S3). Inhibition of viral reverse transcriptase enzyme activity has been previously demonstrated at 140 mM NaCl [17]. The lack of interference with RT-qPCR of nonionic detergents, such as Triton X-100, may be attributable to the stability of MMLV reverse transcriptase or Taq polymerase in Triton X-100-containing buffers [18,19].

TaqMan predesigned hydrolysis assays offer convenience compared to conventional SYBR Green assays, albeit at increased cost per experiment. To improve accessibility and scalability, we considered a reduction in reaction volume, master mix, and primer reagents. With 10 µL reactions, as little as 0.25× master mix and a predesigned assay could be utilized with housekeeping genes without significantly affecting amplification (Figure 1B). We utilized 0.5× master mix and a predesigned assay for all other experiments in this study without loss of amplification.

**Figure 2:**
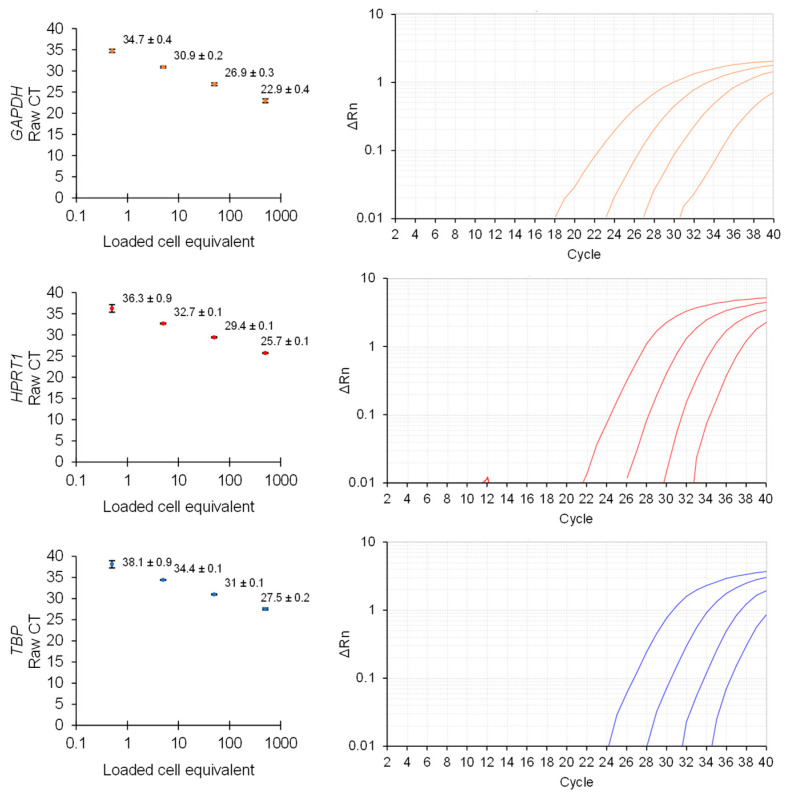
Gene expression population titration of an optimized one-step direct lysis method. The fibroblast cell culture lysate was diluted and analyzed by one-step RT-qPCR for the detection of housekeeper gene expression (*n* = 3, error presented as the standard error of the mean (SEM)) and representative amplification curves.

As the one-step RT-qPCR assay is intended to support microplate format assays with variable cell populations, we established the lowest range of detection by housekeeper genes and found that loading the equivalent lysate extracted from as few as 10–100 human fibroblast cells could reliably provide CT values <35 (Figure 2). The optimized direct lysis RT-qPCR method allows for convenient microplate gene expression analysis at the transcriptional level within 35 min (5 min lysis and 30 min thermocycler time).

### Direct Lysis RT-qPCR Applications

**Figure 3:**
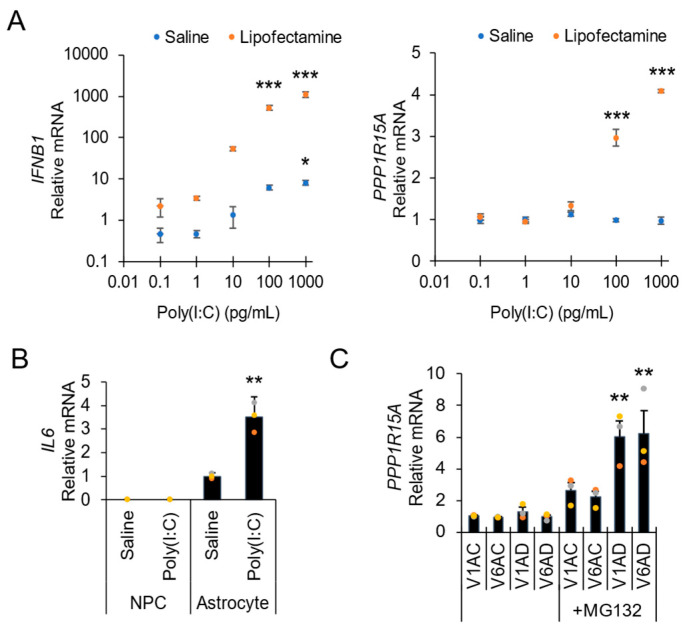
Example applications of rapid direct lysis one-step RT-qPCR assay. (**A**) Application to inflammation studies: dose–response poly(I:C)-induced expression of antiviral interferon (*IFNB1*) and GADD34 (*PPP1R15A*) in human fibroblasts. (**B**) Application to cell differentiation/inflammation studies: stimulation of proinflammatory interleukin 6 (*IL6*) by poly(I:C) in astrocytes compared to neural precursor cells (NPC). (**C**) Application to cell culture disease model: chemical stress-induced GADD34 (*PPP1R15A*) upregulation in eIF2B disease astrocyte lines (V1AD, V6AD) in comparison to non-disease control lines (V1AC, V6AC). Astrocytes were incubated with 0.1 µM MG132 for 24 h (*n* = 3; error presented as the SEM; one-way ANOVA with Holm–Sidak post hoc multiple comparisons test; ** *p* < 0.01, *** *p* < 0.001).

**Figure 4:**
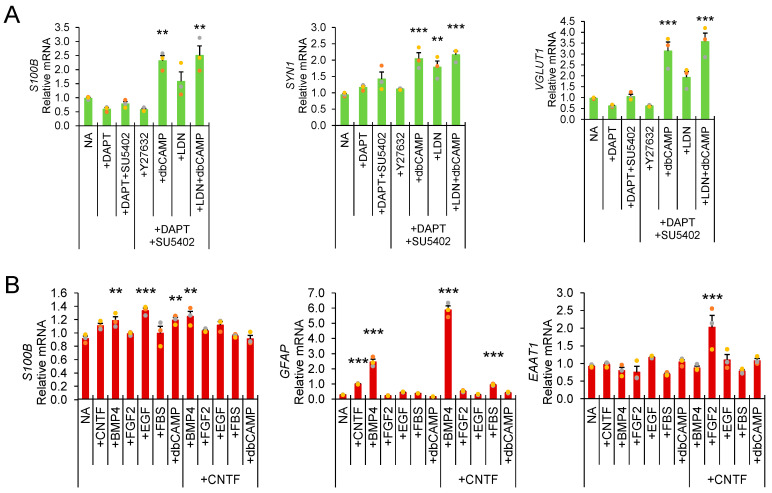
**Example** application of rapid direct lysis RT-qPCR assay for identification of neuronal and astrocyte differentiation factors. (**A**) Neuronal differentiations with combinations of Notch inhibitor DAPT, rock inhibitor Y27632, tyrosine kinase inhibitor SU5402, BMP4 inhibitor LDN-193189 (astroglial differentiation inhibitor), and cyclic AMP agonist db-cAMP. (**B**) Direct lysis RT-qPCR of astrocyte differentiation assays (one-way ANOVA with Holm–Sidak post hoc multiple comparisons test ** *p* < 0.01, *** *p* < 0.001).

To explore the utility of the direct lysis RT-qPCR microplate assay, we applied this protocol to a range of applications, including experiments to investigate gene expression changes during inflammatory signaling (Figure 3A,B), disease/drug mode of action (Figure 3C), and cell differentiation (Figure 4). In the context of inflammatory signaling, the dose–response induction of the inflammatory factor interleukin-6 (*IL6*) and the stress response marker *PPP1R15A* (GADD34) by poly(I:C) indicates intracellular receptor activation of the innate dsRNA immune response of human fibroblasts (Figure 3A). Additionally, the inflammatory function of stem-cell-derived astrocytes based on elevated expression of interleukin-6 (*IL6*) can be compared to neural precursor cells (Figure 3B). Disease and drug mode-of-action can be investigated in small-molecule stress assays, such as with the proteasomal inhibitor MG132 in eIF2B disease astrocytes, which induces differential expression of *PPP1R15A* (Figure 3C).

We also implemented this protocol for the identification of key components of neuronal (Figure 4A) and astrocyte transgene overexpression differentiations from neural precursor stem cells (Figure 4B). In neuronal differentiation assays, small molecules SU5402, db-cAMP, and LDN-193189 increased neuronal culture transcripts of vesicular glutamate transporter 1 (*VGLUT1*) and synapsin 1 (*SYN1*). Db-cAMP also increased early glial marker S100 calcium-binding protein B (*S100B*). Treatment with gamma-secretase inhibitor DAPT [20] increased glutamate-evoked calcium response and neurite outgrowth (Figure S4), without affecting transcript levels of *VGLUT1* or *SYN1*. No modulation of glutamate-induced calcium response, neurite length, or branch number by LDN-193189 or db-cAMP was observed, and similar outcomes were observed with neurotrophic factors BDNF or BDNF and GDNF.

In astrocyte differentiation assays, *S100B* was observed to be upregulated by BMP4, EGF, db-cAMP, and CNTF + BMP4, while CNTF, BMP4, CNTF + BMP4, and FBS upregulated glial fibrillary acidic protein (*GFAP*) expression, and CNTF + FGF2 was associated with upregulated excitatory amino acid transporter 1 (*EAAT1*) expression. CNTF is an established maturity factor commonly utilized in stem-cell-derived astrocyte media and associated with upregulation of GFAP [21], while *EAAT1* (GLT1) has been observed to be upregulated by overnight FGF2 treatment [22].

This protocol can be executed in tandem or multiplex with endpoint gene expression microplate spectroscopy or microscopy-based assays. Potential parallel applications include immunofluorescence and correlation of cell stress/death/maturation transcriptomic markers in cytotoxic/cytoprotective, oxidative stress, mitochondrial content, intracellular calcium activity, wound healing, or neurite outgrowth assays. When multiplexed with a single housekeeper, the assay can be scaled for higher-throughput purposes, such as screening small molecules for cell stress, inflammatory or differentiation markers.

Under basal conditions, cellular stress inducible marker *PPP151RA* (GADD34) was detectable from human fibroblast lysate at a loading of ≥10–100 cells, while quantitative detection of gene expression at the protein level by chemiluminescent Western blots typically requires loading of ≥5 µg (>10,000 cells) (Figure S5). The low cell population requirements of the microplate gene expression assay enables its application as a preliminary screen of gene expression before quantitative confirmation of mature protein expression by lower-throughput Western blots.

This protocol does not include genomic DNA removal, given that the low detergent concentration lysis buffer is unlikely to release nuclear DNA, and primarily relies on the inherent specificity and sensitivity of exon-spanning TaqMan probes. We found that reaction assay mixtures can be prepared and stored at −20 °C for at least several months [23] (Figure S6); however, freezing of the reaction mix with the template resulted in complete loss of amplification. We also utilized samples stored at −80 °C for at least 6 months without deterioration of signal with no additives, as previously published [5] (Figure S6), although we recommend handling of direct lysis samples at 4 °C and avoidance of freeze–thaw cycles. There are a number of limitations to this protocol. Firstly, this protocol was optimized with adherent cell cultures and has yet to be optimized with suspension cultures. Loosely adherent cell cultures should be centrifuged after wash and lysis stages to avoid loss of sample during wash or introduction of cell debris into the lysis buffer. The optimization parameters in this study were restricted to a minimum elongation cycle time of 15 s due to instrumentation limitations; RT-qPCR instruments with faster heating ramp times could reduce thermocycling time well below ≤20 min. Another potential limitation of this protocol is that, while reducing NaCl concentration was found to enable the direct lysis buffer to be applied in the composition suggested for microplate format, other primer pairs not tested in this study may be more adversely affected by saline than others and must be tested before application. Additionally as a direct microplate RT-qPCR assay, only endpoint analysis is feasible, and parallel wells/plates must be set up for multiple timepoints.

## 4. Conclusions

An optimized microplate one-step direct lysis RT-qPCR assay was demonstrated in applications for inflammatory reactivity marker expression, differential patient-derived cell line disease stress marker expression, and neural differentiation cell-based assays. These applications included the intracellular dsRNA inflammatory pathway of activation in human fibroblasts, comparative *IL6* upregulation by NPCs and astrocytes, and stress-induced differential expression of *PPP1R15A* in eIF2B disease patient-derived astrocytes. Key components supporting neuronal maturation (SU5402, db-cAMP, and LDN-193189) and astrocyte maturation (CNTF, BMP4, FGF2, EGF, and db-cAMP) for cell differentiation protocols were identified. With the reduction in reaction volume and concentration to a cost comparable to that of SYBR green assays, as well as decreased run time with multiplex capacity, the protocol can be implemented as an efficient microplate gene expression assay in a cost-effective and scalable manner or in parallel with other cell-based assays.

## Data Availability

Data supporting the findings of this study are available within the article and its Supplementary Materials. Derived data supporting the findings of this study are available from corresponding author on request.

## Supplementary Materials

The following supporting information can be downloaded at: https://www.mdpi.com/article/10.3390/bios12060364/s1.
