# Supplementary material for: An Optimized Direct Lysis Gene Expression Microplate Assay and Applications for Disease, Differentiation, and Pharmacological Cell-Based Studies
Source: Biosensors (Basel). 2022 May 26;12(6):364. doi: 10.3390/bios12060364 (PMC9221485; doi:10.3390/bios12060364)
Supplement: Supplementary file 1 [file biosensors-12-00364-s001.zip › biosensors-1696032-Supplementary.pdf]

An optimized direct lysis gene expression microplate assay and applications for disease, differentiation and pharmacological cell-based studies

Supplementary data

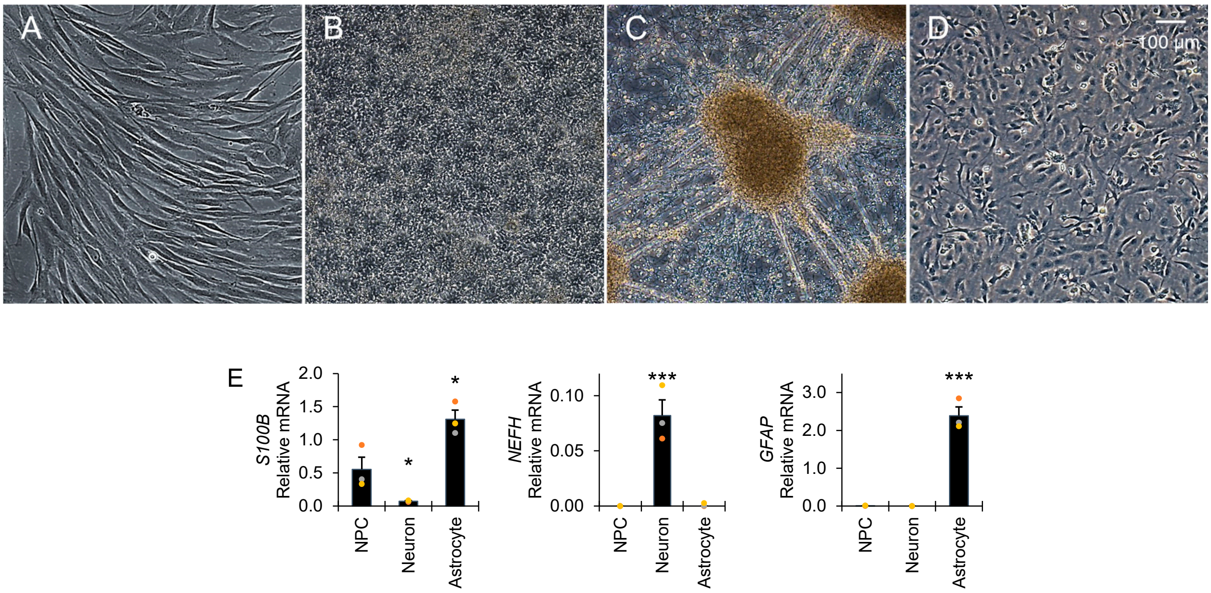

Figure S1. Representative phase contrast images of fibroblast cultures (A), neural precursors (B) neuronal (C) and astrocyte cultures (D) (D14). Neural precursor (NPC) to neuronal and astrocyte differentiations with glial (S100B), astrocyte (GFAP), and neuronal (NEFH), gene expression markers.

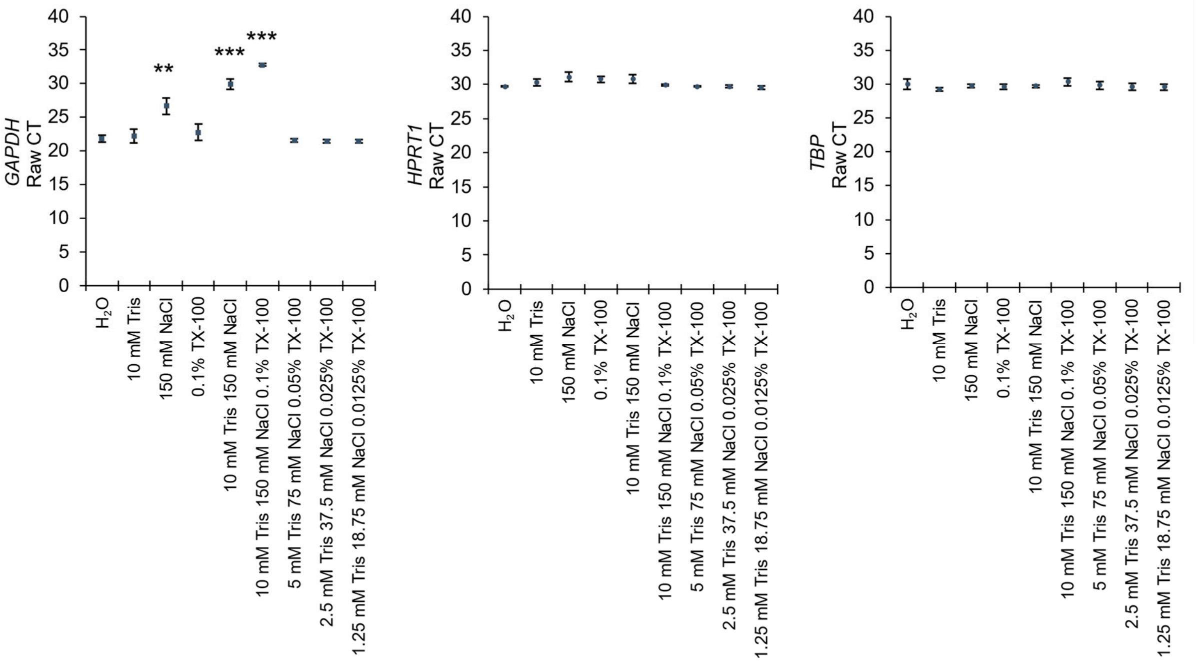

Figure S2. Direct lysis buffer optimization. Amplification of *GAPDH* is inhibited in presence of high NaCl concentration, similar effects noted with other assays targeted to *NAE1* and *IL6* (data not shown) (n = 3; error presented as SEM; ** $p < 0.01$, *** $p < 0.001$).

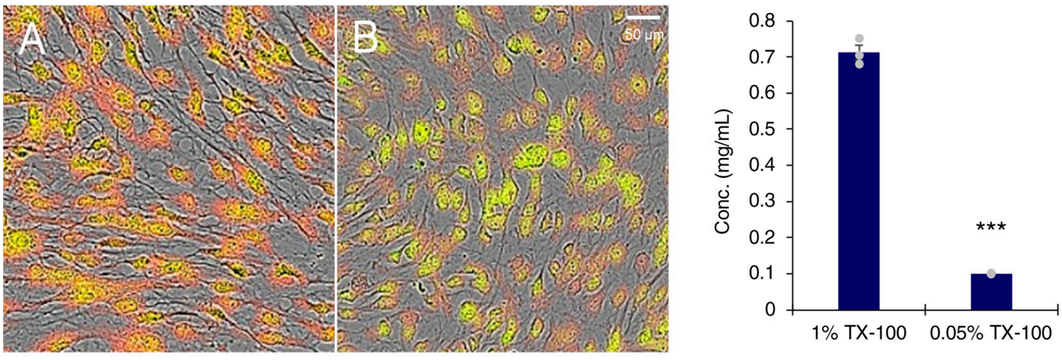

Figure S3. Lysis of cytoplasmic contents but not nuclear DNA evident by cellular protein staining and protein quantification. Trichloroacetic acid fixed and sulforhodamine B staining of cytoplasmic and nuclear protein (red) is present in unlysed control (A) in contrast to altered morphology, decreased cytoplasmic staining but intact nucleus that remains in vessel after wash stage (B). At least >85% of total cellular protein is retained, as demonstrated by BCA quantification of RT-qPCR lysis buffer compared to complete lysis in 1% TX-100.

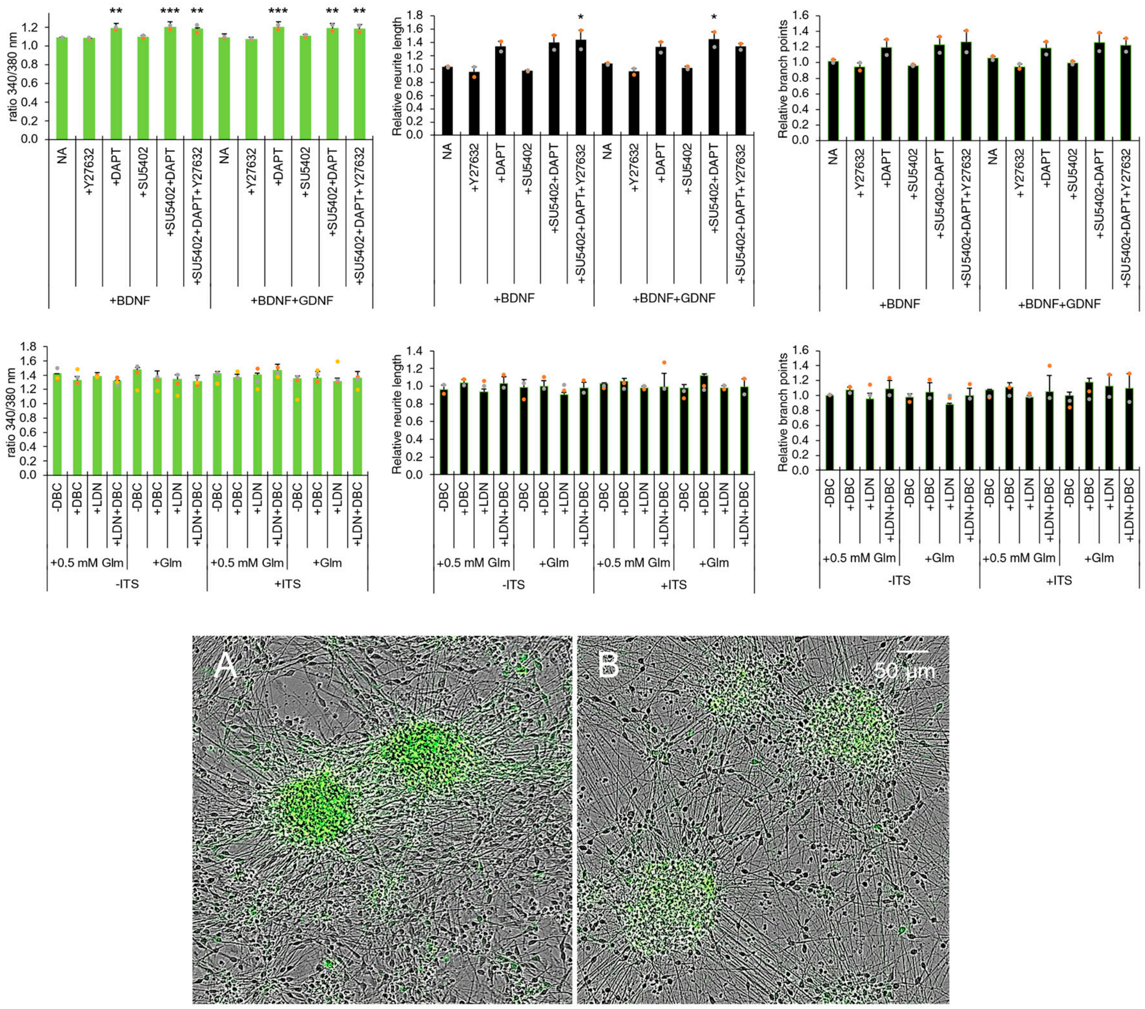

Figure S4. Glutamate calcium assay and neuronal differentiation assay. An increase in glutamate response (Fura-2 AM assay), neurite length and branch point density was observed in cultures treated with 5 µM DAPT. Relative change in neurite length and branch points, glutamate-evoked intracellular calcium assay show similar outcomes between conditions additionally supplemented with ITS, lower or higher levels of GlutaMAX (0.5 mM vs 2 mM), with or without 0.5 mM dbCAMP or 0.1 µM LDN. (A) Neuronal culture morphology in absence or (B) presence of DAPT (one-way ANOVA followed by Holm-Sidak post-hoc multiple comparison, n = 2).

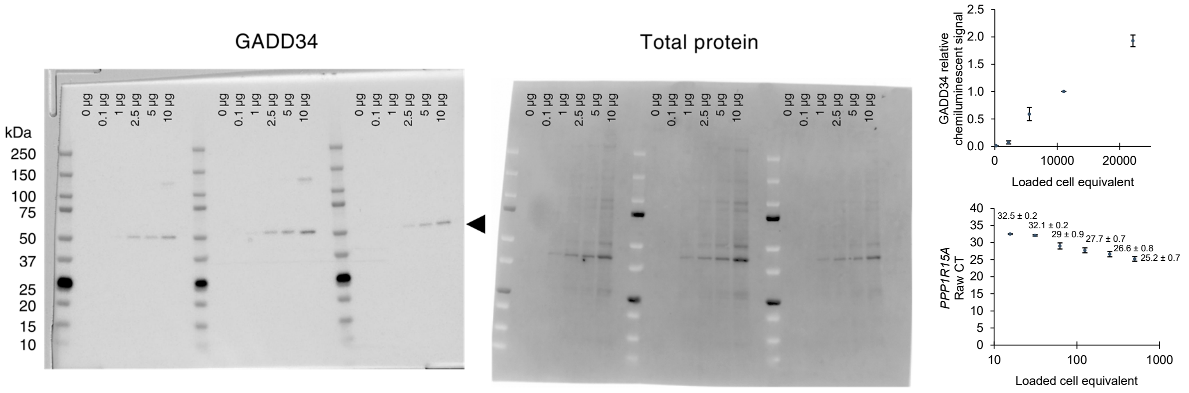

Figure S5. Correlation of chemiluminescent western blot signal to cell lysate loading, and minimal detection of GADD34/PPP1R15A expression at the mRNA and protein level. Human fibroblasts were lysed and processed by enhanced chemiluminescent blot probed for GADD34. Cellular protein visualized by stain-free fluorescent imaging. Western blots were performed as previously described (13). Fibroblast cell cultures were counted using a 0.1 mm Neubauer-improved haemocytometer following dissociation. Protein quantification to permit correlation to cell number was performed using a Pierce BCA Protein Assay Kit (23225, Thermo Fisher Scientific, United States, MA). Briefly, samples were prepared with 4X Laemmli buffer and 2.5% 2-mercaptoethanol, heat denatured at 70 °C for 10 min, and separated by gel electrophoresis at 160 V for 55 min on Criterion TGX Stain-Free Precast Gel 4-20% (5678095, Bio-Rad, United States, CA). Samples were then transferred at 100 V for 1 h on to Amersham Hybond P 0.2 µm PVDF Western blotting membrane (10600021, Amersham, United Kingdom). Membranes were blocked in 5% w/v skim milk for 1 h at ambient temperature before incubation with 1:2000 rabbit anti-GADD34 (2701237, Sigma) in 5% skim milk overnight at 4 °C, before secondary incubation in 1:5000 goat anti-rabbit HRP-conjugated antibody (Thermo Fisher Scientific Scientific G-21234) for 1 h at ambient temperature. Images were viewed with an Amersham Imager 600RGB.

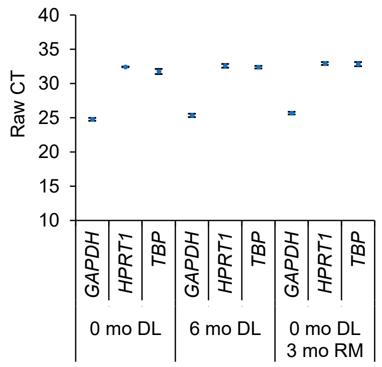

Figure S6. Direct lysis buffer samples following storage at -80 °C or storage of reaction mixture at -20 °C. No significant differences between samples frozen 6 months apart, or with reaction mixture frozen for 3 months.
